# Apolipoprotein E associated with reconstituted high‐density lipoprotein‐like particles is protected from aggregation

**DOI:** 10.1002/1873-3468.13428

**Published:** 2019-05-27

**Authors:** Ellen Hubin, Philip B. Verghese, Nico van Nuland, Kerensa Broersen

**Affiliations:** ^1^ Nanobiophysics Group Technical Medical Centre Faculty of Science and Technology University of Twente Enschede The Netherlands; ^2^ Structural Biology Brussels Department of Biotechnology (DBIT) Vrije Universiteit Brussel (VUB) Belgium; ^3^ Structural Biology Research Center VIB Brussels Belgium; ^4^ Department of Neurology Washington University School of Medicine St. Louis MO USA; ^5^ Applied Stem Cell Technologies Faculty of Science and Technology University of Twente Enschede The Netherlands; ^6^ Present address: C2N Diagnostics Center for Emerging Technologies 4041 Forest Park Ave St. Louis MO 63108 USA

**Keywords:** aggregation, Alzheimer's disease, apolipoprotein E, high‐density lipoprotein, isoform, lipidation

## Abstract

Apolipoprotein E (APOE) genotype determines Alzheimer's disease (AD) susceptibility, with the APOE ε4 allele being an established risk factor for late‐onset AD. The ApoE lipidation status has been reported to impact amyloid‐beta (Aβ) peptide metabolism. The details of how lipidation affects ApoE behavior remain to be elucidated. In this study, we prepared lipid‐free and lipid‐bound ApoE particles, mimicking the high‐density lipoprotein particles found *in vivo*, for all three isoforms (ApoE2, ApoE3, and ApoE4) and biophysically characterized them. We find that lipid‐free ApoE in solution has the tendency to aggregate *in vitro* in an isoform‐dependent manner under near‐physiological conditions and that aggregation is impeded by lipidation of ApoE.

## Abbreviations

**(V)LDL**, (very) low‐density lipoprotein

**AD**, Alzheimer's disease

**ApoE**, apolipoprotein E

**Aβ**, amyloid‐beta peptide

**CD**, circular dichroism

**CSF**, cerebrospinal fluid

**DLS**, dynamic light scattering

**FFF‐MALS**, field flow fractionation multiangle light scattering

**HDL**, high‐density lipoprotein

**MRE**, mean residue ellipticity

**NRMSD**, normalized root mean square deviation

**POPC**, 1‐palmitoyl‐2‐oleoyl‐*sn*‐glycero‐3‐phosphocholine

**SDS/PAGE**, sodium dodecyl sulfate polyacrylamide gel electrophoresis

**TEM**, transmission electron microscopy

**UV**, ultraviolet

Lipids require specialized carriers that transport them through the body, known as apolipoproteins. Apolipoproteins facilitate lipid solubilization and serve as ligands for lipoprotein receptors that mediate cellular lipid uptake and play a role in cell signaling 1. Apolipoprotein E (ApoE) is one of the most studied members of this protein family, as the APOE genotype has been linked to several neurological disorders, with a strong association with Alzheimer's disease (AD) 2, 3. ApoE is produced in abundance in the human brain by astrocytes, in less extent by macrophages and stressed neurons, and is the principal lipid transporter in the cerebrospinal fluid 4.

ApoE exists as three isoforms: ApoE2, ApoE3, and ApoE4 5. The APOE ε4 allele is the most important genetic risk factor for development of late‐onset AD. People carrying one or two copies of the APOE ε4 allele have respectively about 3‐ and 12‐fold more risk of acquiring AD than non‐APOE ε4 carriers 6. In contrast, the APOE ε2 allele is protective 7. ApoE was initially found to colocalize with plaques containing the amyloid‐beta (Aβ) peptide in AD brains 8. Substantial evidence exists that ApoE contributes to AD pathogenesis by modulating Aβ aggregation and clearance, and by regulating brain lipid metabolism and synaptic functioning through ApoE receptors such as those of the low‐density lipoprotein (LDL) receptor family 9, 10, 11, 12. Proposed Aβ‐independent roles for ApoE4 in AD include generation of neurotoxic ApoE fragments, impairment of mitochondrial function, and disruption of the cytoskeleton through stimulation of tau phosphorylation 13.

Although the ApoE isoforms only differ by their amino acid compositions at positions 112 and 158 14, these changes have profound effects on the structure and lipoprotein‐binding preferences of the isoforms 15, 16. ApoE consists of two structural domains linked by a flexible hinge region. Although the N‐ and C‐terminal domains interact in ApoE4, this interaction does not occur to the same extent in ApoE2 and ApoE3 17. The N‐terminal receptor‐binding domain is an extended four‐helix bundle and is responsible for binding to the LDL receptor. The C‐terminal domain of ApoE comprises several amphipathic α‐helices and contains the lipid‐binding region that is capable of binding different types of lipids (e.g., cholesterol, phospholipids, fatty acids) and lipoproteins, including LDLs, very low‐density lipoproteins (VLDLs), and high‐density lipoproteins (HDLs). ApoE in the human brain is mainly synthesized by and secreted from astrocytes to generate ApoE‐containing HDL‐like particles. It has been suggested that astrocyte‐secreted HDL particles are discoidal in shape, but the conformation adopted by ApoE in the lipid complexes remains controversial 16.

The mechanistic link between ApoE and AD has been the subject of numerous studies and debates, but it has become clear that the lipidation status of ApoE plays an important role. For the most part, biologically active ApoE is associated with lipids 18 and the ApoE lipidation status has been reported to impact Aβ metabolism, that is, Aβ aggregation and deposition 19, 20, 21, 22, and clearance 23, 24, 25, 26. For example, enhanced expression of lipidated ApoE in AD mouse models, through activation of liver X receptors or through overexpression of the ATP‐binding cassette A that is responsible for ApoE lipidation, stimulates Aβ clearance 23, 27. Therefore, modulators of ApoE secretion and lipidation are being explored as potential drugs for AD therapy 22, 28, 29.

Studying ApoE behavior in its lipid‐free and lipid‐bound state is thus of great importance to enhance our understanding of its functioning in the context of AD pathology. In this study, we therefore produced all three ApoE isoforms in their lipidated and nonlipidated forms, and systematically characterized and compared them by a range of biophysical techniques. The lipidation procedure was carefully selected to mimic *in vivo* discoidal HDL‐like ApoE particles with a physiological lipid composition consisting of phospholipid and unesterified cholesterol 30, 31, 32. Our results confirm the previously reported tendency of lipid‐free ApoE to self‐assemble in solution 33, 34, 35, 36 and provide experimental evidence that lipidation protects ApoE from aggregation.

## Materials and methods

### Preparation of HDL‐like ApoE particles

#### Preparation of reconstituted ApoE

Lyophilized recombinant human ApoE (Leinco Technologies, Inc., St Louis, MO, USA) was resuspended to a concentration of 1 mg·mL^−1^ in Dulbecco's phosphate‐buffered saline (DPBS, Thermo Fisher Scientific, Landsmeer, The Netherlands) pH 7.4 containing 0.05 mm dithiothreitol.

#### Liposome preparation

1‐Palmitoyl‐2‐oleoyl‐sn‐glycero‐3‐phosphocholine (POPC, Avanti Lipids) and unesterified cholesterol (Avanti Polar Lipids) were mixed in a glass vial at a molar ratio of 90 : 5 and dried under a constant nitrogen gas stream. This ratio was selected to mimic the physiological lipid composition of HDL‐like ApoE particles 30, 31. Lipids were resuspended in PBS at a concentration of 5 μg lipids·μL^−1^ PBS. The solution was mixed thoroughly in a vortex mixer and intermittently for 5–10 min (with 1–2 min intervals) to generate liposomes. Complete hydration of liposomes was accomplished by incubating the solution at room temperature for 30 min and occasional vortex mixing.

#### ApoE lipidation

Lipids can be added directly to ApoE but lipidated particles will be more homogeneous when using the sodium cholate dialysis method 32, 37, 38. Therefore, sodium cholate (50 mg·mL^−1^, Sigma‐Aldrich, St. Louis, MO, USA) was slowly titrated into the liposome solution (2–3 volumes of sodium cholate for 1 volume of lipids). The solution turbidity cleared after 5 min of gentle vortex mixing (1 min interval) and the preparation was kept at room temperature for 30–60 min. Reconstituted ApoE was then added to the liposome preparation (ApoE : POPC : cholesterol, molar ratio of 1 : 90 : 5) and mixed gently for 5–10 min (1–2 min interval). The solution was kept at room temperature for 1 h and dialyzed (10 kDa cutoff membrane) against PBS for 4 h at room temperature (to promote removal of detergents), followed by 60–72 h at 4 °C. After dialysis, samples were analyzed by gel filtration chromatography (Superdex 200 10/300 GL) and nondenaturing (native) polyacrylamide gel electrophoresis (PAGE). ApoE concentrations were determined by absorbance measurements at 280 nm using an extinction coefficient of 44 460 M^−1^·cm^−1^ 39. Samples were diluted in PBS to 0.1 mg·mL^−1^ prior to further analysis. All lipoprotein samples were prepared using the same lipid–cholesterol suspension and the procedure was performed in parallel. Samples were stored at 4 °C.

#### TEM imaging of lipid‐free and lipid‐bound ApoE

A staining procedure was adapted to assess the formation of HDL‐like ApoE particles with TEM 40. Briefly, carbon‐coated Formvar 400‐mesh copper grids (AgarScientific, Stansted, UK) were glow discharged prior to sample application. Lipidated ApoE (2 μL of a 0.1 mg·mL^−1^ sample) was spotted and incubated on the grids for 2 min at room temperature. The grids were subsequently blotted, washed (3 × 2 s, in ultrapure water), and stained with 1% (w/v) uranyl acetate (2 × 2 min). For imaging of lipid‐free ApoE, samples were spotted and incubated on grids for 30 s, blotted, washed (1 × 5 s), and stained with 1% uranyl acetate (1 × 30 s). Samples were studied with a JEM‐1400 microscope (JEOL Ltd., Tokyo, Japan) at 80 kV. Images are representative of at least three independently prepared samples.

#### Native PAGE

Lipoprotein particle formation was assessed by native PAGE. Equal amounts of ApoE isoforms (3 μg) were mixed with Novex^®^ Tris‐Glycine Native Sample Buffer (1 : 1) to obtain a final volume of 15 μL, and loaded on a 4–20% Tris‐glycine gel (Invitrogen). The gel was run at 100 V for 16 h at 4 °C. Sample migration was assessed using the NativeMarkTM Unstained protein standard (Life Technologies).

#### FFF‐MALS

For each fractionation, a volume of 10 μL ApoE (0.1 mg·mL^−1^) was injected in an Eclipse asymmetrical flow field flow fractionation (FFF) system (Wyatt Technology, Santa Barbara, CA, USA), and the flow rate out of the channel was maintained at 1 mL·min^−1^. Fractionated samples were analyzed with multiangle light scattering (MALS) using the DAWN HELEOS system (Wyatt Technology), an ultraviolet (UV) detector, and an Optilab rEX refractive index detector (Wyatt Technology) connected to the Eclipse system. The MALS system was equipped with a laser operating at 658 nm and measurements were taken at 14.4°, 25.9°, 34.8°, 42.8°, 51.5°, 60.0°, 69.3°, 79.7°, 90.0°, 100.3°, 110.7°, 121.2°, 132.2°, 142.5°, 152.5°, and 163.3°, with reference to the axis of the incident beam. astra v software (version 5.3.4.14) (Wyatt Technology, Santa Barbara, CA, USA) was used for data acquisition and correction for interdetector delay and band broadening.

#### DLS

Lipid‐free and lipid‐bound ApoE (0.1 mg·mL^−1^ in PBS) were analyzed using dynamic light scattering (DLS). DLS experiments were conducted with a DynaPro DLS plate reader (Wyatt Technology) at 25 °C and at a scattering angle of 158°. Data were analyzed using Dynamics^®^ software (Wyatt Technology) and represent the averages of 15 acquisitions (10 s per acquisition).

#### CD

ApoE isoforms (0.1 mg·mL^−1^ in PBS) in the absence and presence of lipids were placed in a quartz cuvette with an optical path of 0.1 cm. Far‐UV circular dichroism (CD) spectra were recorded in a Jasco J‐715 spectropolarimeter (Jasco, Tokyo, Japan) at 25 °C. The wavelength range was set from 260 to 190 nm with 0.2‐nm resolution, 8.0‐s response time, and 1.0‐nm bandwidth. Data were collected as averages of eight scans at a scanning speed of 50 nm·min^−1^. Spectra were corrected by subtracting the buffer baseline. Measurements were performed as independent duplicates. Data are presented as the mean residue ellipticity (MRE, in deg cm^2^·dmol^−1^). Secondary structure content was estimated using cdsstr software and the normalized root mean square deviation (NRMSD) is displayed as a measure of correspondence between the experimental and calculated reference spectra 41, 42.

#### Intrinsic tryptophan fluorescence

Emission fluorescence spectra of lipidated and nonlipidated ApoE isoforms (0.1 mg·mL^−1^ in PBS) were measured using a LS 55 spectrometer (PerkinElmer, Waltham, MA, USA) at 25 °C. The excitation wavelength was set to 280 nm (5 nm bandwidth) and the emission intensity was scanned from 300 to 450 nm (5 nm bandwidth) at a scan speed of 100 nm·min^−1^. Spectra were corrected for buffer and represent averages of eight scans. Measurements were performed as independent duplicates.

## Results

**Figure 1:**
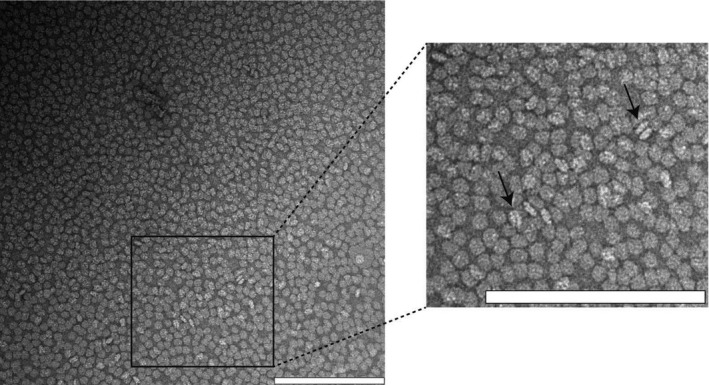
Assessment of the formation of HDL‐like discoidal ApoE particles with TEM. The majority of the discoidal ApoE particles are visualized from their top/bottom, but some can also be seen from a lateral perspective (indicated by arrows). The scale bars represent 200 nm. The image is representative of at least three independently prepared samples.

Astrocyte‐secreted ApoE in the brain is predominantly associated with cholesterol and phospholipid‐rich HDL‐like complexes 30, 31. Therefore, HDL‐like ApoE particles were prepared using 1‐palmitoyl‐2‐oleoyl‐*sn*‐glycero‐3‐phosphocholine (POPC) and unesterified cholesterol, in a 1 : 90 : 5 molar ratio (ApoE : POPC : cholesterol), using the sodium cholate dialysis method described previously 38. The lipidation procedure was assessed by transmission electron microscopy (TEM) and revealed discoidal lipidated ApoE particles (Fig. 1).

**Figure 2:**
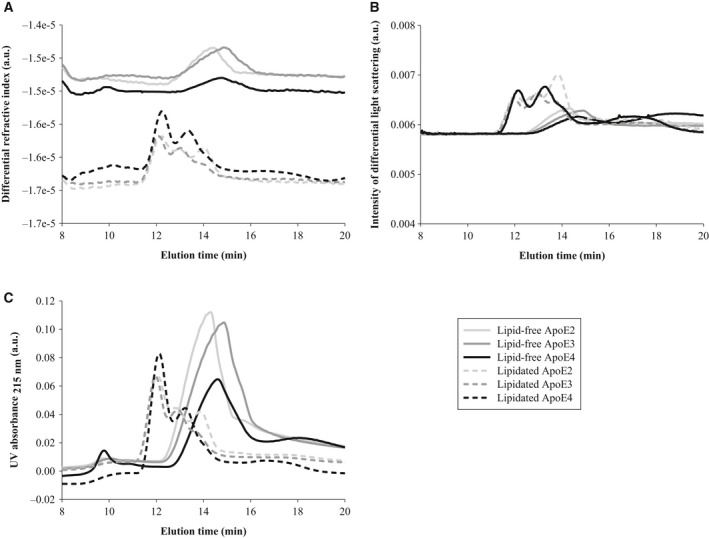
The heterogeneous composition of HDL‐like ApoE particles. Lipid‐free and HDL‐like ApoE particles (0.1 mg·mL^−1^ in PBS) were separated by FFF and their composition was compared by their (A) differential refractive index, (B) intensity of differential light scattering, and (C) UV absorbance at 215 nm. Obtained spectra are representative of two independently prepared ApoE isoform samples.

The sodium cholate procedure resulted in a heterogeneous population of lipid‐bound ApoE particles, as shown by field flow fractionation multiangle light scattering (FFF‐MALS) analysis that detected three fractions with different retention times (Fig. 2). FFF is a high‐resolution separation technique that consists of a velocity gradient inside a channel that separates particles based on their size. Smaller particles will be more rapidly transported through the channel than larger ones and will elute first, as opposed to size‐exclusion chromatography. The heterogeneity detected for lipidated ApoE particles is consistent with previous studies reporting different sizes for ApoE‐containing lipoproteins secreted by astrocytes from transgenic mice expressing human ApoE, and in cerebrospinal fluid (CSF) of human subjects 31, 43, 44.

**Figure 3:**
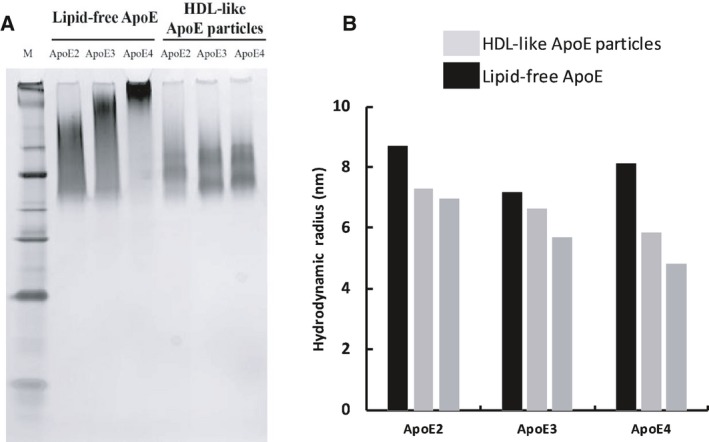
Lipidation impedes aggregation of ApoE. Migration patterns and size distributions of lipid‐free and HDL‐like ApoE particles (0.1 mg·mL^−1^ in PBS) were obtained by native PAGE and DLS, respectively. (A) Lipidated ApoE migrates further in a 4–20% Tris‐glycine gel compared to lipid‐free ApoE (M: NativeMarkTM Unstained protein standard). (B) The hydrodynamic radius of lipidated ApoE is smaller than that of lipid‐free ApoE. Obtained data are representative of two independently prepared ApoE isoform samples.

Next, ApoE isoforms in their lipid‐free and lipid‐bound state were characterized using FFF‐MALS, native polyacrylamide gel electrophoresis (PAGE), and dynamic light scattering (DLS). The first particles to elute from the FFF channel were the HDL‐like ApoE particles, and not the lipid‐free ApoE isoforms, as detected by differential refractive index analysis (Fig. 2A), MALS (Fig. 2B), and UV absorbance (Fig. 2C). Although lipid‐free ApoE was eluted around 15 min, lipidated ApoE particles displayed shorter retention times, that is, between 12 and 14 min. This result indicates that the size of lipidated ApoE, and specifically the hydrodynamic radius, is smaller than that of lipid‐free ApoE. Accordingly, native PAGE revealed that lipid‐bound ApoE migrated further in the 4–20% Tris‐glycine gel than lipid‐free ApoE (Fig. 3A). Moreover, estimations of the hydrodynamic radii by DLS confirmed that lipidated ApoE, regardless of the ApoE isoform, was smaller than lipid‐free ApoE (Fig. 3B). Together, these results suggest that lipid‐free ApoE has the tendency to aggregate in solution at a concentration of 0.1 mg·mL^−1^, whereas lipidation is capable of impeding this behavior. This tendency is isoform dependent, with the most pronounced aggregation for ApoE4, followed by ApoE3 and ApoE2 (Fig. 3).

**Figure 4:**
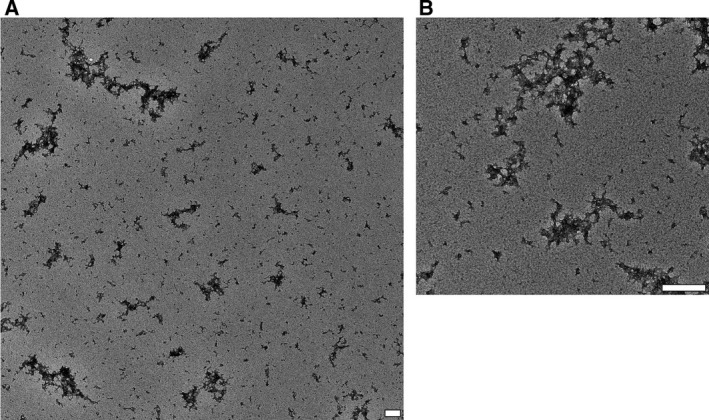
Lipid‐free ApoE4 self‐assembles into amorphous aggregates. (A) Lipid‐free ApoE aggregates displayed an amorphous morphology, similar for all three isoforms, as assessed by TEM. Lipid‐free ApoE4 aggregates are depicted. (B) An enlarged image of lipid‐free ApoE4 aggregates. The scale bars represent 200 nm. Images are representative of at least three independently prepared samples.

The aggregation of lipid‐free ApoE4 was visualized by TEM and revealed amorphous aggregates (Fig. 4).

**Figure 5:**
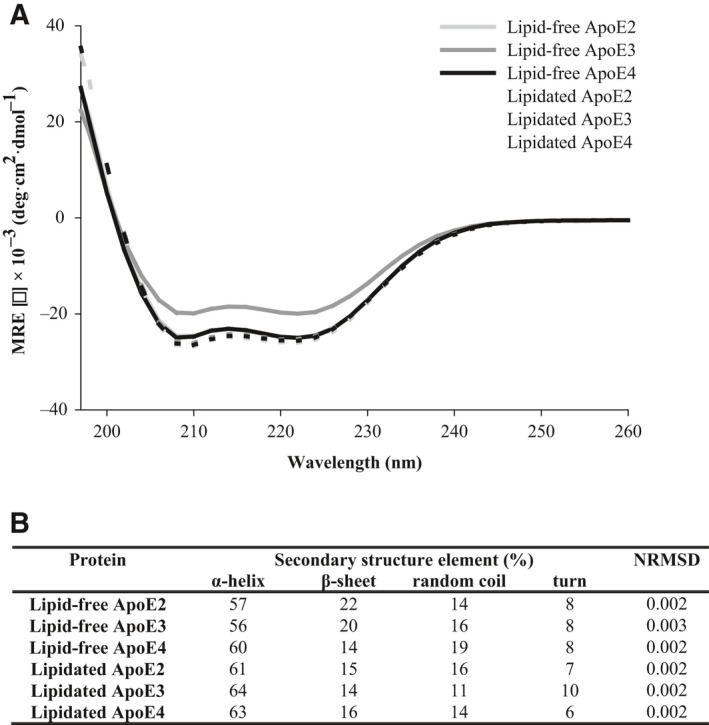
Effect of lipidation on the secondary structure of ApoE. The secondary structure content of lipid‐free and HDL‐like ApoE particles (0.1 mg·mL^−1^ in PBS) was studied by CD. (A) CD reveals a predominant α‐helical structural signature for all samples characterized by double minima around 208 and 222 nm. (B) Secondary structure content of each sample was estimated using cdsstr software 41, 42. The goodness of fit of the experimental CD data with the reference data is indicated by the NRMSD value. Spectra are averages of two independently prepared duplicates.

To assess the effect of lipidation on secondary structure content of ApoE, circular dichroism (CD) measurements were performed. Lipid‐free as well as lipid‐bound ApoE displayed a predominant α‐helical structural signature, characterized by two minima around 208 and 222 nm (Fig. 5A). Lipid‐free and lipidated ApoE displayed approximately 60% α‐helicity (Fig. 5B), which corresponds to values reported previously 45. The mean residue ellipticity was, however, slightly increased in the lipidated ApoE state with a small gain of α‐helicity and loss of β‐sheet structure (Fig. 5B). However, taken into account an approximate error of 5% in the measurements, the overall effect of lipidation on the secondary structure of ApoE was minor.

**Figure 6:**
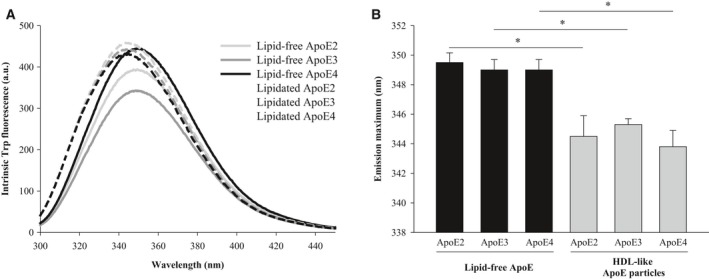
Effect of lipidation on the tertiary structure of ApoE. (A) Intrinsic Trp fluorescence emission spectra (λex = 280 nm) corresponding to lipid‐free and HDL‐like ApoE particles (0.1 mg·mL^−1^ in PBS). (B) The maximum of the Trp fluorescence emission spectrum of lipidated ApoE is blue shifted compared to that of lipid‐free ApoE. Statistical significance of the results was established by *P*‐values using unpaired two‐tailed *t*‐tests, with *P < 0.05. Spectra are averages of two independently prepared duplicates.

In contrast, more pronounced differences could be observed in terms of tertiary structure, when lipid‐free and lipid‐bound ApoE were compared by their intrinsic Trp fluorescence. ApoE has seven Trp residues: four are located in the N‐terminal domain and three are situated in the C‐terminal lipid‐binding domain. ApoE particles displayed a marked blue shift in their fluorescence maximum upon lipidation (Fig. 6). We attribute this blue shift to tertiary structural alterations in the vicinity of the Trp residues resulting in an increased hydrophobic environment.

## Discussion

ApoE has been reported to self‐assemble 33, 34, 35, 36 and the hypothesis has been raised that the amphipathic α‐helical structure of ApoE is stabilized upon lipid binding, which may protect it from amyloidogenic folding pathways 36. We provide experimental evidence that lipidation indeed impedes aggregation of ApoE, by comparing lipid‐free ApoE and HDL‐like discoidal ApoE particles of all three ApoE isoforms using a biophysical approach.

Our results show that lipid‐free ApoE has the tendency to self‐assemble, with ApoE4 having the highest aggregation propensity, followed by ApoE3 and ApoE2 (Figs 2, 3, 4). This is in accordance with previous observations that provide evidence that ApoE oligomerizes through a monomer–dimer–tetramer association process 34, and can aggregate further from tetramers to higher molecular weight aggregates 33, 35. These aggregates displayed an α‐helical structure, in accordance with our results (Fig. 5) 36. Moreover, the ApoE aggregation rate was previously shown to be isoform dependent (ApoE4 > ApoE3 > ApoE2), which was attributed to differences in conformational stability of the ApoE N‐terminal region, with a decreased stability resulting in a higher aggregation rate 36. Not only ApoE but also other apolipoproteins including ApoA‐I, ApoA‐II, and ApoB100 display low conformational stability and have the tendency to self‐assemble 46.

Despite the importance of the stability of the N terminus, several studies have appointed the C terminus as the main determinant of ApoE self‐assembly 35, 47, 48, 49, 50. The C terminus of ApoE comprises amphipathic α‐helices and exposes a large, hydrophobic surface 17. As the lipid‐binding region of ApoE is situated in the C‐terminal region of ApoE, it was hypothesized that there might be a link between ApoE self‐assembly and its lipid‐binding properties 51, 52. Accordingly, we provide experimental evidence that lipidation impedes ApoE self‐assembly into amorphous aggregates, as ApoE bound to lipids is smaller than when alone in solution, based on its hydrodynamic radius and migration properties (Figs 2, 3, 4). Lipidation has minor effects on the secondary structure of ApoE, with the main contribution still arising from α‐helices (Fig. 5), but elicits tertiary structural alterations in the vicinity of Trp residues (Fig. 6). This observation is consistent with the general consensus that ApoE undergoes a lipid binding‐induced conformational rearrangement 16. It has been suggested that lipidation might stabilize the amphipathic α‐helical structure of ApoE and protect it against aggregation 36. This is not a property solely applicable to ApoE but also to other apolipoproteins that contain a large proportion of amphipathic α‐helices and display low conformational stability in the absence of lipids 46. It is conceivable that variations in lipid composition may influence the observations made, as it has been shown that distinct discoidal ApoE lipid complexes are formed when varying POPC and cholesterol levels 53. Further studies are required to establish the impact of levels and type of surface and core lipids on aggregation of ApoE.

Although ApoE mostly occurs in its lipid‐bound form in plasma and CSF, there are lipid‐poor reservoirs and conditions that are vulnerable to aggregation (e.g., ApoE synthesized by macrophages and neurons in stress conditions) 36. Therefore, the higher propensity of lipid‐free ApoE4 in solution to aggregate compared to other ApoE isoforms as shown by our data, and its ability to form aggregates that are toxic to neuronal cells 36, might underlie its association with AD.

In conclusion, we report that lipid‐free ApoE in solution has the tendency to aggregate *in vitro* in an isoform‐dependent manner (ApoE4 > ApoE3 > ApoE2) under near‐physiological conditions and that aggregation is impeded by lipidation of ApoE. As ApoE4 aggregates have been demonstrated to be toxic to neuronal cells, these findings might explain the higher AD risk associated with ApoE4 in comparison with other ApoE isoforms.

## Author contributions

The manuscript was written through contributions of all authors. KB and NvN conceived and supervised the study; KB, NvN, and PV designed experiments, EH, KB, and PV performed experiments, PV provided new tools, EH, NvN, KB, and PV analyzed data. All authors have given approval to the final version of the manuscript.

## Funding

This work was supported by an FWO doctoral fellowship (EH), the VIB and the Flemish Hercules Foundation (NvN), Internationale Stichting Alzheimer Onderzoek (ISAO) (KB), an FWO Odysseus II award (KB) and a UTWIST fellowship (KB).
